# Alteration of Loperamide-Induced Prostate Relaxation in High-Fat Diet-Fed Rats

**DOI:** 10.1155/2014/517836

**Published:** 2014-11-20

**Authors:** Sheng-Lung Hsu, Hsien-Hui Chung, I-Hung Chen, Yat-Ching Tong

**Affiliations:** ^1^Department of Urology, Chi-Mei Medical Center Jiali, Jiali Town, Tainan City, Taiwan; ^2^Institute of Basic Medical Sciences, College of Medicine, National Cheng Kung University, Tainan City, Taiwan; ^3^Institute of Clinical Medical Sciences, College of Medicine, National Cheng Kung University, Tainan City, Taiwan; ^4^Department of Urology, Medical College and Hospital, National Cheng Kung University, Tainan City, Taiwan; ^5^National Cheng Kung University Hospital, 138 Sheng-Li Road, Tainan City 704, Taiwan

## Abstract

*Objective*. To investigate the change of loperamide-induced prostate relaxation in rats fed with high-fat diet (HFD). *Materials and Methods*. Adult male Wistar rats were divided into 2 groups: (1) control rats fed with normal chow and (2) rats fed with HFD for 6 months. The prostate was removed for histology study. Isolated prostate strips were hung in organ bath and precontracted with 1 *μ*mol/L phenylephrine or 50 mmol/L KCl. The relaxation responses to loperamide 0.1 to 10 *μ*mol/L were recorded. Western blotting analyses were performed for prostate *μ*-opioid receptors (MOR) and ATP-sensitive potassium (K_ATP_) channel proteins: sulfonylurea receptor (SUR) and inwardly rectifying potassium channel (Kir) 6.2 subunits. *Results*. Body weight, prostate weight, plasma levels of glucose, insulin, triglyceride, and cholesterol, as well as systolic blood pressure, were significantly increased in the HFD rats. Histology showed prostatic hyperplasia in the HFD rat prostate. Prostatic relaxation induced by loperamide was markedly reduced in HFD when compared to the control. Protein expressions of MOR, SUR, and Kir 6.2 were decreased in HFD-fed rats. *Conclusion*. Loperamide-induced prostate relaxation is decreased in HFD rats due to reduced MOR and K_ATP_ channel expressions.

## 1. Introduction

There are two important mechanisms leading to benign prostate obstruction (BPO): a static component due to an increase in prostate size (volume) and a dynamic component due to an increase in prostate tone (contraction). Over the last four decades, neurophysiological study on prostate contractility has been focused on the sympathetic alpha-adrenergic function [1]. As such, the alpha-blockers have been the mainstay drug treatment for clinical benign prostatic hyperplasia (BPH). However, a recent animal study on the rat has shown the presence of *μ*-opioid receptors (MOR) in the prostate and its role in inducing relaxation through ATP-sensitive potassium (K_ATP_) channels [2]. Thus the neural network controlling prostate contraction is probably more complicated than we used to know.

On the other hand, the knowledge of etiological factors that may lead to BPH has also increased in recent years. Epidemiology data have shown positive correlations between obesity, metabolic syndrome, and the occurrence of lower urinary tract symptoms (LUTS) suggestive of BPH [3]. Metabolic syndrome is a combination of risk factors for cardiovascular diseases, which include obesity, insulin resistance, hypertension, and dyslipidemia [4]. In the Third National Health and Examination Survey (NHANES III), men classified as having three or more components of the metabolic syndrome had increased odds of LUTS [5]. In animal studies, it was demonstrated that metabolic syndrome in fructose-fed obese rats decreased tissue perfusion, induced glandular hyperplasia, and increased smooth muscle contraction of the prostate [6, 7]. In addition, high-fat diet (HFD) feeding in rats induced insulin resistance that led to increased cellular proliferation, enhanced alpha-adrenoceptor mediated contraction, and enlargement of the prostate [8].

Loperamide is an MOR agonist which does not cross the blood-brain barrier [9]. The drug can induce prostate relaxation through its action on the MOR [2]. The present study aimed to investigate the effect of HFD on the MOR-mediated prostate relaxation in the rat. Loperamide-induced prostate relaxation responses in normal chow-fed and HFD-fed rats were compared. In addition, the possible mechanisms involving MOR and K_ATP_ channel expressions underlying the differences were investigated.

## 2. Materials and Methods

### 2.1. Experimental Animals

Twelve-week-old male Wistar rats (body weight 350 to 400 g) were kept in a temperature-controlled room (25 ± 1°C) with 12 h light-dark cycle (lights on at 06:00). The rats were given water and food* ad libitum* and were divided into two groups according to the diet. One group was fed with standard laboratory diet (3.04 kcal/g). The other group was fed with HFD (5.16 kcal/g) (TestDiet, Richmond, IN, USA) for 6 months. The study protocol was approved by institutional animal care and use committee. All animal-handling procedures were performed according to the* Guide for the Care and Use of Laboratory Animals* of the National Institutes of Health, as well as the guidelines of the Animal Welfare Act.

### 2.2. Measurement of Metabolic Parameters

The body weight, blood pressure, plasma glucose, insulin, cholesterol, and triglyceride levels were measured before and after the 6-month feeding period. Systolic blood pressure was measured with a noninvasive tail-cuff monitor (MK2000; Muromachi Kikai, Tokyo, Japan). Eight-hour fasting plasma glucose was measured with a Beckman Glucose Analyzer. Plasma insulin was determined with a radioimmunoassay kit (Linco Research, Inc., St. Charles, MO). The plasma cholesterol and triglyceride levels were measured using enzymatic methods (Roche, Pleasanton, CA, USA) through an automatic analyzer (Roche).

### 2.3. Preparation of Isolated Prostate Strips

The method we used for rat prostate strip contractile response study had been previously reported [2, 10]. The rat was sacrificed by decapitation under anesthesia with intraperitoneal pentobarbital (50 mg/kg). The ventral prostate was surgically removed via a midline abdominal incision and immediately placed in oxygenated (95% O_2_, 5% CO_2_) Krebs' solution at 37°C containing (in mmol/L) NaCl 135; KCl 5; CaCl_2_ 2.5; MgSO_4_ 1.3; KH_2_PO_4_ 1.2; NaHCO_3_ 20; and d-glucose 10 (pH 7.4). Tissue strips about 10 × 5 mm were fashioned longitudinally from the prostate and mounted in organ bath filled with 10 mL oxygenated Krebs' solution. Each preparation was connected to strain gauges (FT03; Grass Instrument, Quincy, MA, USA). Isometric tension was recorded using Chart Software (MLS023, Powerlab; ADInstruments, Bella Vista, NSW, Australia). Strips were gradually stretched to achieve an optimal resting tension of 0.5 g and allowed to stabilize for 2 h.

### 2.4. Prostatic Relaxation Induced by Loperamide

Solution of either phenylephrine (Sigma-Aldrich, St. Louis, MO, USA) or KCl prepared in distilled water was added to the organ bath to induce a rapid phasic contraction followed by a sustained tonic contraction of the prostate strips. The final concentration for phenylephrine (PE) was 1 *μ*mol/L and for KCl was 50 mmol/L. During the plateau phase of the tonic contraction, loperamide (0.1–10 *μ*mol/L) was added to induce relaxation of the strips. Relaxation was expressed as the percentage tension decrease from the maximum tonic contraction. Concentration-relaxation curves were generated in cumulative fashion.

### 2.5. Histology Study

The rat prostate was removed and fixed in 10% formaldehyde at 4°C for 2 days. Fixed specimens were dehydrated and embedded in paraffin. The specimens were cut into 5 *μ*m thick sections at 50 *μ*m intervals and then stained with hematoxylin and eosin (H&E; Muto Pure Chemicals, Tokyo, Japan). The sections were observed under a light microscope. Tissue strips that had been previously used for contractile studies were not reused for histology or Western blotting experiments.

### 2.6. Western Blotting Analysis

The prostate tissues were put in ice-cold homogenized buffer containing 10 mM Tris-HCl (pH 7.4), 20 mM EDTA, 10 mM EGTA, 20 mM *β*-glycerolphosphate, 50 mM NaF, 50 mM sodium pyrophosphate, 1 mM phenylmethylsulfonyl fluoride, and the protease inhibitors 25 *μ*g/mL leupeptin and 25 *μ*g/mL aprotinin. The mixture was centrifuged at 1000 ×g for 10 min at 4°C. The supernatant containing the membrane fraction was centrifuged at 48,000 ×g for 30 min at 4°C. The supernatant was removed, and the pellet was resuspended in Triton X-100 lysis buffer on ice for 30 min, homogenized, and then centrifuged at 14,010 ×g for 20 min at 4°C. Finally, the supernatant was transferred to a new Eppendorf tube and stored at −80°C. The membrane extracts (20–80 *μ*g) were separated by performing SDS-polyacrylamide gel electrophoresis, and the proteins were transferred onto a BioTraceTM polyvinylidene fluoride (PVDF) membrane (Pall Corporation, Pensacola, FL). Following blocking, the blots were developed using antibodies for MOR (Abcam, Cambridge, UK), sulfonylurea receptor (SUR) (Millipore), or inwardly rectifying potassium channel (Kir) 6.2 subunits (Kir 6.2) (Santa Cruz Biotechnology, CA). The blots were subsequently hybridized using horseradish peroxidase-conjugated goat anti-goat IgG (Jackson ImmunoResearch Laboratories, Inc., PA) and developed using the Western Lightning Chemiluminescence Reagent PLUS (PerkinElmer Life Sciences Inc., Boston, MA). Densities of the obtained immunoblots at 48 KDa for MOR, 170 KDa for SUR, 40 KDa for Kir 6.2, and 43 KDa for actin were quantified using Gel-Pro analyzer software 4.0 (Media Cybernetics, Silver Spring, MD, USA). Comparisons of protein expression levels between the two groups of rats were given in “arbitrary unit” which was calculated by dividing receptor expression density by that of actin.

### 2.7. Statistical Analysis

All values are presented as the mean ± standard error of the mean (SEM) for the number (*n*) of animals or individual experiments. Statistical analysis was performed using the IBM SPSS Statistics software. One-way analysis of variance (ANOVA) and Dunnett's post hoc test were used to compare the loperamide-induced relaxation responses between the HFD and control rats. Student's *t*-test was used to compare the metabolic parameters and protein expression levels between the two groups. A *P* value of less than 0.05 was considered as statistically significant.

## 3. Results

### 3.1. The Effects of HFD on Rat Body Weight, Blood Pressure, and Metabolic Parameters

Table 1 compares the body weight, systolic blood pressure, metabolic parameters, and the prostate weight between the HFD and control rats. Manifestations of metabolic syndrome including obesity, hyperglycemia, hyperinsulinemia, hyperlipidemia, and hypertension were observed in the HFD rats.

### 3.2. Prostate Changes in HFD Rats

In contrast to the control, the prostates of HFD rats were significantly enlarged with increased weights (Table 1). Histology study showed glandular hyperplasia with closely packed small acini and intraglandular papillae in the HFD rat prostate.

### 3.3. Alteration of Loperamide-Induced Prostate Relaxation in HFD Rats

**Figure 1:**
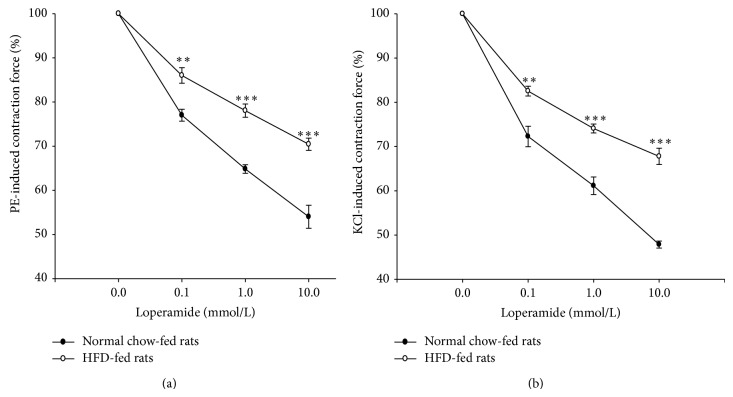
Concentration-dependent relaxation induced by loperamide in isolated prostate strips precontracted with 1 *μ*mol/L phenylephrine (a) or 50 mmol/L KCl (b) in control and HFD rats, respectively. Data represent mean ± SEM of eight individual experiments. ^**^ *P* < 0.01, and ^***^ *P* < 0.001 compared with the control group.

Precontraction with either PE (1 *μ*mol/L) or KCl (50 mmol/L) produced similar biphasic phasic-tonic contractile responses of the prostatic strips. As shown in Figure 1(a), loperamide relaxed PE-contracted prostate strips from control and HFD rats in a concentration-dependent manner. The effect of loperamide was reversible after washout and repeatable with a second application. Compared with the control, the relaxation effect of PE-induced prostate contraction by loperamide in HFD rats was significantly reduced. Similarly, the loperamide-induced relaxation in prostatic strips precontracted by KCl was also reduced in the HFD rats (Figure 1(b)).

### 3.4. Alterations of Protein Expressions in the HFD Rat Prostate

**Figure 2:**
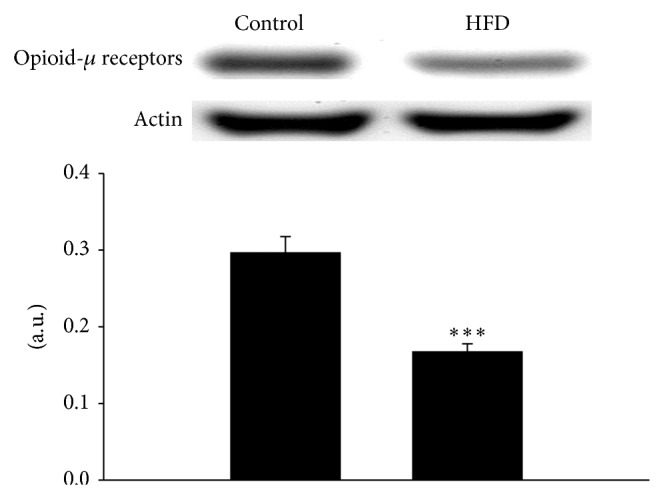
The difference in the protein levels of *μ* opioid receptors (MOR) between control and HFD rat prostate. Western blot densities for opioid *μ*-receptors were corrected with actin as internal standard. Data represent mean ± SEM of six individual experiments. ^**^ *P* < 0.01 compared with the control group.

**Figure 3:**
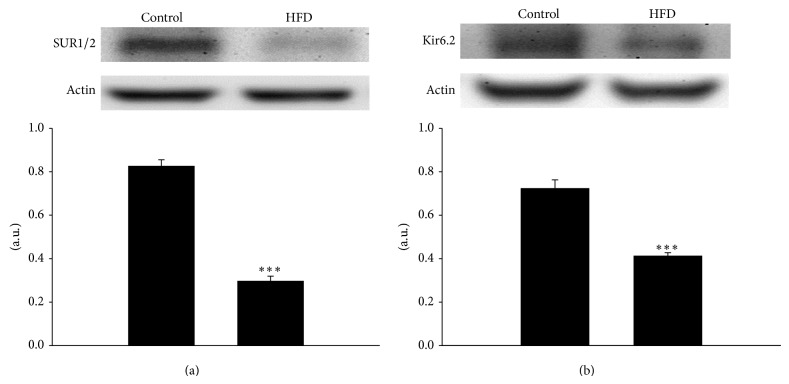
The difference in the protein levels of sulphonylurea receptor isoforms 1 and 2 (SUR1/2) (a) and inwardly rectifying K^+^ channel subunit 6.2 (Kir 6.2) (b) between control and HFD rat prostate. Actin was used as internal standard. Data represent mean ± SEM of six individual experiments. ^***^ *P* < 0.001 compared with the control group.

As shown in Figure 2, the expression of opioid *μ*-receptors in the HFD rat prostate was significantly lower than that in normal chow-fed rats. On the other hand, the expressions of SUR1/2 and Kir 6.2 in HFD rat prostates were also significantly decreased as compared with controls (Figure 3).

## 4. Discussion

Opiate drugs, like heroin, exert their effects through binding to a group of specific receptor sites called opioid receptors. There are endogenous opioids including endorphins, endomorphins, enkephalins, dynorphins, and nociceptin. The opioid receptors are classified into four main subtypes: mu (*μ*), kappa (*κ*), delta (*δ*), and the nociceptin receptor [11]. Morphine was the first ligand found to bind to MOR, so the subtype was named after the initial letter “M” of the drug. The majority of the MOR are distributed in the central nervous system. Activation of these MOR can cause analgesia, sedation, nausea, euphoria, miosis, hypotension, and suppression of respiration. In addition, MOR are found in the intestinal tract. Activation of the intestinal MOR results in inhibition of peristalsis. However, the role of opioid receptors in controlling lower urinary tract (LUT) function has only been reported in a few studies. Tramadol, an opioid receptor agonist, significantly increased threshold pressure and micturition volume in rat cystometry [12]. U-50488, a kappa opioid agonist, could decrease detrusor-sphincter dyssynergia and improve voiding efficiency in spinal cord injured rats [13]. However, these effects on the LUT were attributed to actions in the central nervous system. Earlier evidence has suggested opioid peptides and opiergic neurons in association with neuroendocrine cells in the human prostate [14, 15]. More recently, protein expression of MOR was demonstrated by Western blotting in the rat prostate and pharmacological activation of the receptor resulted in prostate relaxation [2]. Yet, the identity of the endogenous binding ligands on the prostate MOR is still unclear.

The current study demonstrated prostatic hyperplasia associated with a decrease of MOR expression in the HFD rat. Therefore, the receptor not only can play a role in prostate contractile physiology, but also may be involved in prostate pathophysiology: linking obesity with LUTS. Theoretically, decreased relaxation means increased contraction of the prostate, a key element leading to BPO. Thus the HFD rat model can mimic human BPH in two aspects, increased prostate volume due to glandular hyperplasia and increased prostate contractility due to reduced MOR-mediated relaxation.

Opioid receptors are G protein-coupled receptors that sense molecules outside the cell and activate intracellular signal transduction pathways and subsequently cellular responses. There are two principal signal transduction pathways involving the G protein-coupled receptors: the cyclic AMP (cAMP) signal pathway and the phosphatidylinositol signal pathway. A prior study showed that the mechanism for loperamide-induced prostatic relaxation was mediated through cAMP-protein kinase A (PKA) pathway to open K_ATP_ channels [16]. The activation of K_ATP_ channels causes hyperpolarization of cell membrane and consequently smooth muscle relaxation. K_ATP_ channels are heterooctameric complexes consisting of four pore-forming subunits (inwardly rectifying potassium channel, subfamily J; Kir6.x) and four regulatory sulfonylurea receptor subunits (ATP-binding cassette protein, subfamily C; SURx) [17]. Two Kir6.x (Kir6.1 and Kir6.2) and two SURx (SUR1 and SUR2) subunits have been identified, and their various combinations can give rise to different functional K_ATP_ channel subtypes [18]. In the present study, expressions of SUR1/2 and Kir 6.2 were both decreased in the HFD-fed rat prostate, thus indicating a decrease of K_ATP_ channel expression. Taken together, the decreased loperamide-induced prostate relaxation in HFD rats can be explained by the combined effects of reduction in MOR and K_ATP_ channel expression.

Loperamide is a phenylpiperidine derivative exhibiting affinity and selectivity for the intestinal MOR. The drug exerts antidiarrheal action by decreasing bowel peristalsis and fluid secretion, as well as increasing fluid and electrolyte absorption [19]. Because of its low oral absorption and inability to cross the blood-brain barrier, it has minimal central nervous system effects. In another study, we have demonstrated that the drug can induce bladder muscle relaxation in the rat also through activation of MOR [20]. However, its effects on human lower urinary tract function have rarely been reported. In an anecdotal report, a 10-year-old girl suffered from prolonged urinary retention after receiving oral loperamide for an acute gastroenteritis [21]. In the future, if we could extrapolate these results on rats to human, drugs like loperamide may be potentially useful for BPO and overactive bladder because of the relaxation effects on the prostate and bladder. On the other hand, the findings in the current study suggest that the effectiveness of loperamide in treating BPO in patients with obesity or metabolic syndrome may be reduced possibly due to reduced MOR and K_ATP_ channel expression. Thus further studies in the human are required to investigate the role of MOR agonists in the treatment of lower urinary tract symptoms.

## 5. Conclusion

The current study demonstrated that loperamide-induced prostate relaxation was decreased in HFD rats due to reduced MOR and K_ATP_ channel expression. The findings may be useful in understanding the pathophysiology linking obesity and BPO.

## Figures and Tables

**Table 1:** Body weight, prostate weight, systolic blood pressure (SBP), and metabolic parameters in normal chow-fed control rats and high-fat diet-fed rats.

	Control	HFD
Body weight (g)	456.13 ± 7.12	719.88 ± 8.30^***^
Prostate (g)	0.39 ± 0.01	0.73 ± 0.02^***^
Glucose (mg/dL)	98.00 ± 2.36	137.25 ± 2.60^***^
Insulin (*μ*U/mL)	18.63 ± 1.53	38.45 ± 5.98^**^
SBP (mmHg)	112.75 ± 2.10	146.13 ± 1.82^***^
Cholesterol (mg/dL)	43.88 ± 5.57	119.63 ± 7.16^***^
Triglyceride (mg/dL)	62.25 ± 11.86	146.38 ± 7.43^***^

HFD: high-fat diet.

Values represent the mean ± SEM of eight animals. ^**^ *P* < 0.01, ^***^ *P* < 0.001 compared with the control group.
